# Structure of diaminohydroxyphosphoribosylaminopyrimidine deaminase/5-amino-6-(5-phospho­ribosylamino)uracil reductase from *Acinetobacter baumannii*

**DOI:** 10.1107/S174430911301292X

**Published:** 2013-05-23

**Authors:** Alice Dawson, Paul Trumper, Georgios Chrysostomou, William N. Hunter

**Affiliations:** a Division of Biological Chemistry and Drug Discovery, College of Life Sciences, University of Dundee, Dundee DD1 5EH, Scotland

**Keywords:** bifunctional deaminase/reductase, *Acinetobacter baumannii*, RibD, riboflavin biosynthesis

## Abstract

The bifunctional diaminohydroxyphosphoribosylaminopyrimidine deaminase/5-­amino-6-(5-phosphoribosylamino)uracil reductase (RibD) represents a potential antibacterial drug target. The structure of recombinant *Acinetobacter baumannii* RibD is reported in orthorhombic and tetragonal crystal forms at 2.2 and 2.0 Å resolution, respectively. Comparisons with orthologous structures in the Protein Data Bank indicated close similarities. The tetragonal crystal form was obtained in the presence of guanosine monophosphate, which surprisingly was observed to occupy the adenine-binding site of the reductase domain.

## Abstract 2

The structure of a bifunctional deaminase/reductase involved in riboflavin biosynthesis in the pathogen *A. baumannii* has been determined in two crystal forms.

## 1. Introduction

*Acinetobacter* species are a significant and increasingly important cause of hospital-acquired infections (Gaynes & Edwards, 2005 ▶), owing in part to a remarkable ability to resist antibiotic challenge (Peleg *et al.*, 2008 ▶). Some strains are resistant to almost all available antibiotics, leading to an urgent need for novel treatments for this pathogen. As part of a wide-ranging project to advance early-stage drug discovery against *Pseudomonas aeruginosa* and closely related Gram-negative organisms such as *A. baumannii* (Eadsforth *et al.*, 2012 ▶; Moynie *et al.*, 2013 ▶), we have adopted a structure-based approach to drive the assessment of potential targets (Hunter, 2009 ▶). We identified that several genes encoding enzymes in the riboflavin-biosynthetic pathway are essential in Gram-negative bacteria such as *P. aeruginosa* (see, for example, Liberati *et al.*, 2006 ▶). This was supported by single gene knockout studies in the closely related *A. baylyi* (de Berardinis *et al.*, 2008 ▶). Riboflavin is a precursor for the biosynthesis of flavins, which are essential cofactors for a diverse range of key redox reactions (Macheroux *et al.*, 2011 ▶). Animals rely on dietary sources to acquire riboflavin, while bacteria generally synthesize it *de novo*. This essential aspect of bacterial metabolism is absent in mammals and therefore suggests that this biosynthetic pathway might represent a potential source of antimicrobial drug targets (Mack & Grill, 2006 ▶; Long *et al.*, 2010 ▶). We selected a bifunctional enzyme, diaminohydroxyphosphoribosylaminopyrimi­dine deaminase (EC 3.5.4.26)/5-amino-6-(5-phosphoribosylamino)­uracil reductase (EC 1.1.1.193), called RibD, in this pathway as a potential target in Gram-negative bacteria.

**Figure 1:**
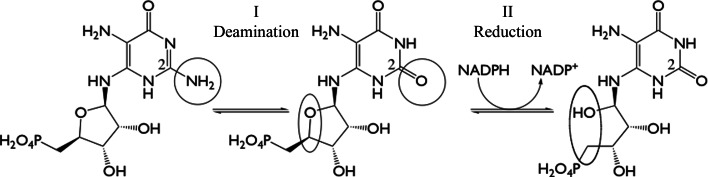
The reactions catalyzed by RibD. The sites of deamination and reduction are circled.

Riboflavin synthesis is a multistep pathway starting from guanosine triphosphate (GTP; Fischer & Bacher, 2005 ▶; Abbas & Sibirny, 2011 ▶). In bacteria, the second and third stages of this metabolic process involve zinc-dependent deamination and an NADPH-dependent reduction at the ribosyl group, respectively (Fig. 1 ▶). The bifunctional enzyme RibD, termed RibG in some bacteria such as *Bacillus subtilis*, catalyzes these two reactions. Fungi and archaea possess two monofunctional enzymes instead of a bifunctional RibD/RibG system.

RibD is constructed from an N-terminal deaminase domain, which carries a Zn^2+^ ion, and a C-terminal NADPH-binding reductase domain. There is no evidence of crosstalk between the domains, as replacement of the bifunctional RibD by monofunctional enzymes in *Escherichia coli* still gave detectable turnover. In addition, knockout of the deaminase activity by mutagenesis of the zinc-binding residues did not affect the reductase activity (Magalhães *et al.*, 2008 ▶).

Structures of the bifunctional enzymes from *E. coli* (*Ec*RibD; Stenmark *et al.*, 2007 ▶), *B. subtilis* (*Bs*RibG; Chen *et al.*, 2006 ▶, 2009 ▶) and *Thermotoga maritima* (PDB entry 2hxv; Joint Center for Structural Genomics, unpublished work) have been reported. Structures of the monofunctional reductases from *Methanocaldococcus jannaschii* (Chatwell *et al.*, 2006 ▶) and *Shewanella* sp. (PDB entry 3ky8; Joint Center for Structural Genomics, unpublished work) have been deposited in the PDB. The structure of *Ec*RibD has been reported in the apo form and in complex with NADPH and ribose-5-phosphate, while that of *Bs*RibG has been reported in the apo form, with NADPH and in complex (separately) with the products of the reductase and deaminase domains. All of these complexes were obtained by crystal soaking and the ligands are present at low occupancy or in only one of several subunits in the asymmetric unit.

We sought to generate a recombinant source of RibD to investigate its structure and to obtain a template for early-stage drug discovery. Initially, we worked with *P. aeruginosa* RibD, but did not obtain crystals despite extensive screening (data not shown). However, recombinant *A. baumannii* RibD (*Ab*RibD) was obtained in high yield and the ordered crystals enabled us to derive structures in two crystal forms.

## 2. Materials and methods

### 2.1. Protein expression and purification

The *A. baumannii ribD* gene was amplified by PCR from genomic DNA (strain ATCC 19606) using 5′-**CTCGAG**ATGTCTGAGTTAAAACAAGATCAATACTGG-3′ and 5′-**GGATCC**TCATACTTTCTCTTGCGTAGG-3′ as the forward and reverse primers, respectively. These oligonucleotides include 5′ *Xho*I and 3′ *Bam*HI restriction sites, respectively, which are indicated in bold. The PCR product was ligated into pCR-Blunt II-TOPO (Invitrogen) and then subcloned into a modified pET15b vector (Novagen), which produces an N-­terminally histidine-tagged protein with a *Tobacco etch virus* (TEV) protease site. The hexahistidine tag, TEV cleavage site and additional residues from the choice of cloning sites added 24 amino acids at the N-terminus. The untagged protein consists of 362 amino acids and the tagged sample consists of 386 residues.

*Ab*RibD was produced using *E. coli* strain BL21(DE3) (Stratagene). Cells were grown in 1 l Luria–Bertani medium supplemented with 50 µg ml^−1^ carbenicillin. Gene expression was induced at 293 K using 1 m*M* isopropyl β-d-1-thiogalactopyranoside and growth continued for 16 h at room temperature. The cells were harvested by centrifugation for 25 min (4000*g* at 277 K), resuspended in lysis buffer (50 m*M* Tris–HCl pH 7.5, 250 m*M* NaCl, 20 m*M* imidazole) containing DNase I (0.1 mg) and a single tablet of EDTA-free protease-inhibitor cocktail (Roche) and lysed using a French press at 110 MPa. Insoluble debris was separated by centrifugation (39 000*g* for 25 min at 277 K) and the soluble fraction was loaded onto a 5 ml HisTrap HP column (GE Healthcare) pre-charged with Ni^2+^. A linear concentration gradient was applied to the column and *Ab*RibD eluted in two peaks at concentrations of 80 and 150 m*M* imidazole. Fractions were analyzed using SDS–PAGE; the two peaks were kept separate during the following steps. Attempts to remove the His tag resulted in precipitation of the protein; therefore, this was avoided and the sample was further purified using a Superdex 200 26/60 size-exclusion column (GE Healthcare) equilibrated with 50 m*M* Tris–HCl, 250 m*M* NaCl pH 7.5. Selected fractions were pooled, dialyzed into 20 m*M* Tris–HCl, 50 m*M* NaCl pH 7.5 and concentrated to 10 mg ml^−1^ using Amicon Ultra devices (Millipore). The protein concentration was determined spectrophotometrically using a theor­etical extinction coefficient of 31 775 *M* ^−1^ cm^−1^ at 280 nm calculated using *ProtParam* (Gasteiger *et al.*, 2005 ▶) based on just the 362 amino acids. The extinction coefficient at 280 nm for all 386 residues was estimated to be 33 265 *M* ^−1^ cm^−1^ and that assuming that all cysteine residues were reduced was 32 890 *M* ^−1^ cm^−1^. These values affected the second decimal place of the concentration, which we consider to be insignificant given the other sources of inaccuracy. The high level of sample purity was verified by matrix-assisted laser desorption/ionization time-of-flight (MALDI–TOF) mass spectrometry and SDS–PAGE (data not shown).

### 2.2. Crystallization and data collection

Crystallization trials were carried out by sitting-drop vapour diffusion with a Rigaku Phoenix automated dispenser and commercially available screens. In addition to the apo form, attempts were made to crystallize binary complexes with NADPH or guanosine monophosphate (GMP) by incubating the protein with the ligands at 5 m*M*, which is about a 20-fold molar excess, for 1 h prior to setting up trials. Initial hits were optimized using hanging-drop vapour diffusion and the best crystals of the apo protein (form I) were obtained with the reservoir conditions 0.9 *M* ammonium phosphate, 0.075 *M* sodium acetate pH 4.5 from drops consisting of 1 µl protein solution and 1 µl reservoir solution. Crystals were obtained from the sample containing GMP (form II) with the reservoir conditions 0.2 *M* ammonium sulfate, 0.125 *M* sodium cacodylate pH 5, 30% polyethylene glycol 8000. No crystals were observed in the presence of NADPH. For reasons that are unclear, crystals were only obtained from the sample comprising the second peak from the Ni^2+^ column. Large block-shaped crystals, which were later shown to represent two crystal forms, typically appeared at 293 K within a few days. The crystals attained dimensions of approximately 0.3 × 0.2 × 0.2 mm.

Prior to data collection, the crystals were briefly soaked in a sample of reservoir solution adjusted to contain 30%(*v*/*v*) glycerol. The other components of this solution were therefore diluted. The crystals were then mounted on a goniostat and maintained at 100 K in a flow of cooled nitrogen, and their diffraction properties were characterized using a Rigaku MicroMax-007 rotating-anode generator with an R-­AXIS IV^++^ image plate. Diffraction data were collected in-house at a wavelength of 1.5418 Å, integrated using *XDS* (Kabsch, 2010 ▶) and scaled with *SCALA* (Evans, 2006 ▶).

### 2.3. Structure solution, refinement and modelling

A search model for molecular replacement based on *Ec*RibD (PDB entry 2g6v; Stenmark *et al.*, 2007 ▶), which shares 46% sequence identity with *Ab*RibD, was prepared in which nonconserved side chains were truncated to alanine using *CHAINSAW* (Stein, 2008 ▶). The structure of form I, with two molecules in the asymmetric unit, was solved using *Phaser* (McCoy *et al.*, 2007 ▶). The two molecules were treated independently during refinement. Rounds of model manipulation using *Coot* (Emsley & Cowtan, 2004 ▶) interspersed with refinement using *REFMAC*5 (Murshudov *et al.*, 2011 ▶) were used to complete the protein model with the addition of water molecules, Zn^2+^ and phosphate ions and the incorporation of dual conformers. Translation–libration–screw (TLS) refinement using three domains per subunit was added in the final stages of refinement; domain assignment was carried out using the *TLSMD* server (Painter & Merritt, 2006 ▶). The first TLS domain spans the entire N-terminal domain, while the C-­terminal domain was divided into two TLS segments. The second crystal form was solved by molecular replace­ment (*Phaser*) using a subunit from crystal form I as the search model and was refined using the strategy described for form I, with the addition of sulfate in place of phosphate and the additional ligands cacodylate, oxalate and GMP. Crystallographic statistics are presented in Table 1 ▶.

## 3. Results and discussion

### 3.1. General comments and overall structure

A highly efficient *E. coli* recombinant expression system for *Ab*RibD was constructed and a purification protocol was established that provided in excess of 100 mg per litre of culture. Two species of the protein were identified in the first purification step using Ni^2+-^-affinity chromatography, only one of which gave rise to crystals. We are unable to explain these observations. The expected mass of a subunit of the tagged construct is 42 038 Da and the samples appeared to be identical on SDS–PAGE, with an approximate mass of 40 kDa, and displayed a similar mass in MALDI–TOF mass spectrometry (42 441 and 42 485 Da). Both samples also appeared to be homogeneous samples of approximate mass 58 kDa in gel filtration and we judge it likely that this species represents an RibD dimer. The overall structure of *Ab*RibD is an arch-like dimer and most certainly not a uniform globular entity. Such an unusual assembly may explain the reduction in apparent mass when analyzed by size-exclusion gel chromatography.

**Figure 2:**
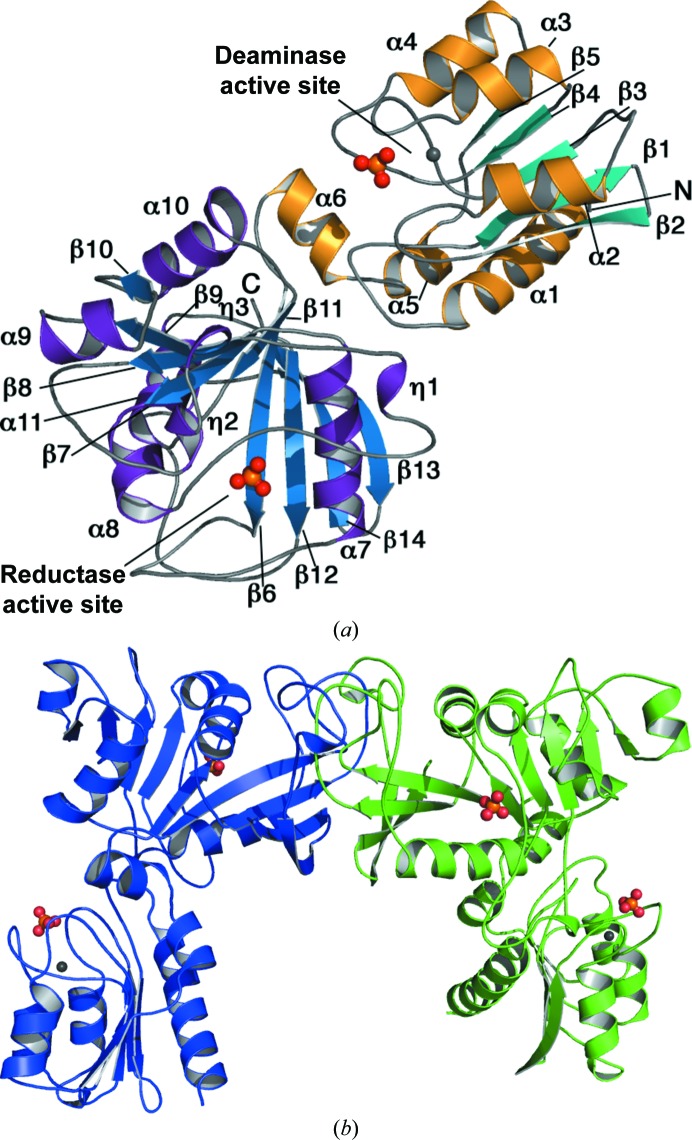
(*a*) The *Ab*RibD subunit structure. Secondary-structure elements are coloured as follows: blue β-­strands and purple helices for the reductase domain and cyan β-­strands and gold helices for the deaminase domain. The Zn^2+^ ion is indicated by a grey sphere and the phosphate ion by orange (P) and red (O) spheres. (*b*) The *Ab*RibD dimer. The subunits are coloured green and blue and the active sites are indicated by the phosphate and Zn^2+^ ions.

Two distinct crystal forms of *Ab*RibD were obtained. Crystal form I, the apoenzyme, belonged to the orthorhombic space group *C*222_1_ with two subunits (*A* and* B*) in the asymmetric unit. Each subunit consists of two domains (Fig. 2 ▶ *a*). The two subunits are related by a noncrystallographic twofold axis (Fig. 2 ▶ *b*). The second form, which crystallized in space group *P*4_3_2_1_2, has one subunit in the asymmetric unit, with a crystallographic twofold generating the same dimer as observed in form I. Both crystal forms have an estimated Matthews coefficient of 3.2 Å^3^ Da^−1^ and a solvent content of about 60%.

The N-terminal reductase domain spans residues 1–150 (coincident with the first TLS domain in both crystal forms) and the C-terminal domain spans residues 151–361. No electron density was observed for the residues forming the N-terminal His tag in either crystal form. Continuous electron density was observed for residues 2–359 in chain *A* and residues 2–358 in chain *B* for crystal form I. Residues 173 and 174 could not be modelled in form II, which was otherwise complete from residues 2 to 357.

**Figure 3:**
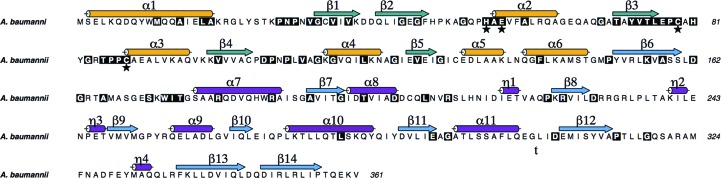
Sequence conservation in RibD. A total of 550 sequences annotated as both EC 3.5.4.26 and EC 1.1.1.193 in the UniProt database (http://www.uniprot.org/) were filtered at 90% sequence identity and aligned using *MUSCLE* (Edgar, 2004 ▶). Residues that are conserved in at least 70% of the 550 sequences are shown on a black background. The Zn^2+^-binding residues are marked with a star and assigned secondary-structure elements are shown with the same colour scheme as used in Fig. 2 ▶. This figure was prepared using *ALINE* (Bond & Schüttelkopf, 2009 ▶).

The N-terminal domain consists of a five-stranded mixed β-sheet flanked by three α-helices on one side (α2, α3 and α4) and two α-­helices (α1 and α5) on the other. A short α-helix (α6) forms the linker to the C-terminal domain, which contains a central nine-stranded predominately parallel β-sheet (strand β14 is antiparallel) with a pronounced twist. Two helices (α7 and α8 on one side and α10 and α11 on the other) lie on each side of the sheet, in addition to several long loops with short 3_10_-helical or η-helical segments and one further α-helix (α9) (Fig. 2 ▶ *a*). The secondary structure is matched onto the primary structure in Fig. 3 ▶.

Chains *A* and *B* overlay with an r.m.s.d. of 0.74 Å over 353 aligned C^α^ atoms. This indicates a high degree of similarity, especially considering that noncrystallographic restraints were not applied during refinement. The largest differences arise in the positions of two loops, one involving residues 106–113 close to the active site of the deaminase domain and the other involving residues 167–174 close to the reductase active site. These differences are described in more detail below. Chain *A* of the apoprotein overlays with the unique chain of form II with an r.m.s.d. of 0.92 Å over 349 aligned C^α^ positions and the dimer of form I overlays with the dimer (generated by the operation of a crystallographic twofold) of form II with an r.m.s.d. of 1.5 Å over 691 aligned C^α^ atoms.

A chain of apo *Ec*RibD superimposes on chain *A* of apo *Ab*RibD with an r.m.s.d. of 1.4 Å over 321 aligned C^α^ atoms, while *Bs*RibG and *Ab*RibD align with an r.m.s.d. of 2.0 Å over 327 C^α^ atoms. This degree of similarity is commensurate with the levels of sequence identity. *Ab*RibD shares 46% sequence identity with *Ec*RibD and 40% with *Bs*RibG.

A number of ligands are included in the crystallographic models; with one exception, these were deliberate additions. The exception is oxalate, which was observed in crystal form II. Oxalate is a common contaminant of polyethylene glycols owing to polymer breakdown (Jurnak, 1986 ▶) and crystal form II was obtained in the presence of polyethylene glycol 8000. We made a similar observation previously in the analysis of PurB (Fyfe *et al.*, 2010 ▶).

### 3.2. The oligomeric state

*Ec*RibD is dimeric (Stenmark *et al.*, 2007 ▶) and *Bs*RibG is tetrameric (Chen *et al.*, 2006 ▶). Analysis of the *Ab*RibD crystal structures using the *PISA* (*Protein Interfaces, Surfaces and Assemblies*) server (Krissinel & Henrick, 2007 ▶) suggests that the crystallographic dimer of *Ab*RibD (Fig. 2 ▶ *b*) is of biological relevance. The interface is formed solely by residues in the C-terminal domain, burying approximately 11% of the surface area (∼1930 Å^2^) of each subunit (in both crystal forms). The interface is formed primarily by a long stretch of residues from Val312 to Leu354, consisting of a long loop joining strands β12 and β13 and of parts of β8 and β9. An additional stretch of residues Ser159–Ile174 is also involved at the interface. 23 hydrogen bonds are formed at the interface, which is significantly larger in area with more interactions than the comparable interface observed in *Ec*RibD (interface area of ∼1310 Å^2^ with only three hydrogen bonds). One subunit–subunit interface in *Bs*RibG is similar to that of *Ab*RibD, burying approximately 2000 Å^2^ per subunit and forming 20 hydrogen bonds*.*

### 3.3. The deaminase domain

**Figure 4:**
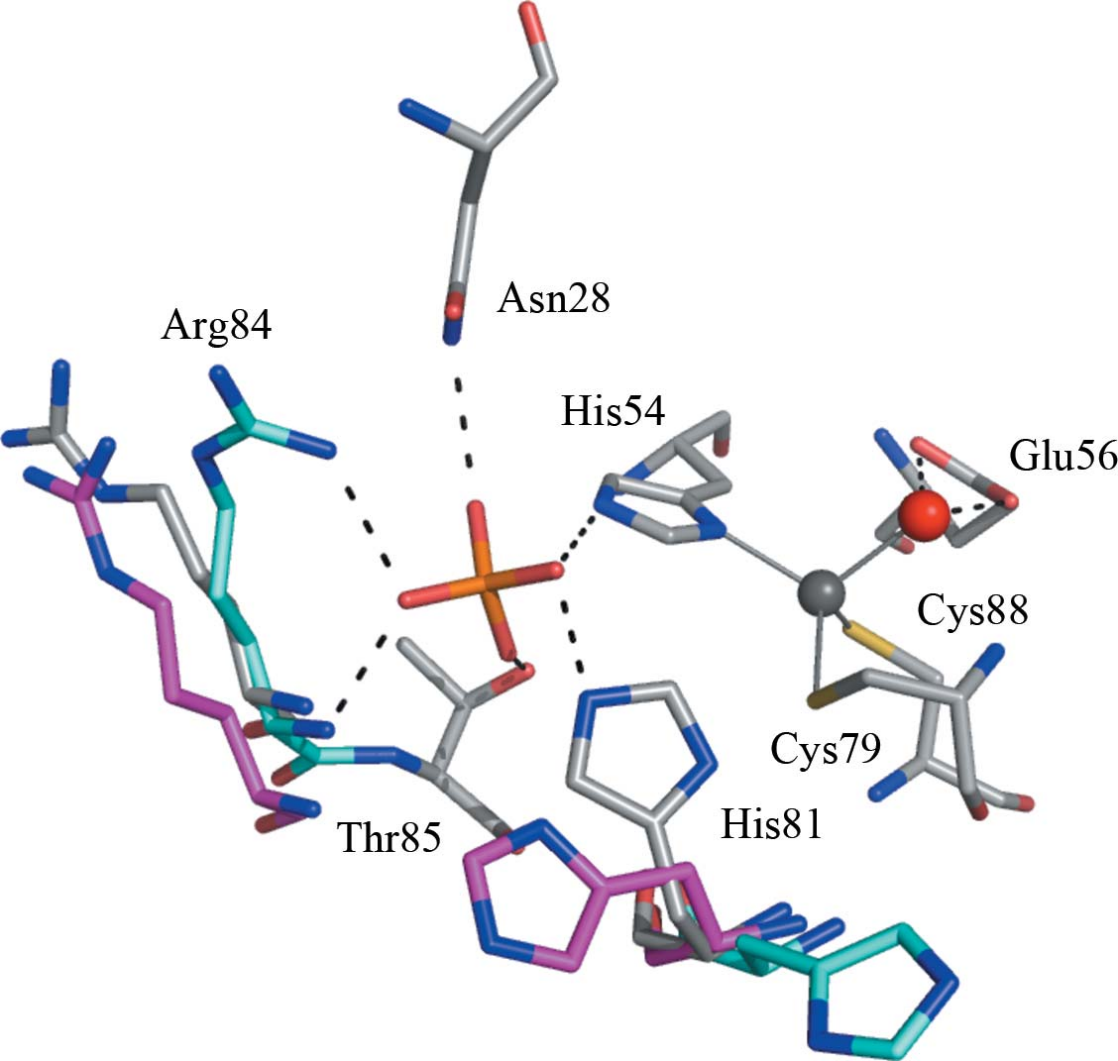
The coordination of Zn^2+^ and oxyanion binding in the deaminase active site. Residues involved in coordination are shown in stick representation: C, grey; N, dark blue; O, red; P, orange; S, yellow. Residues with grey C atoms indicate subunit *A* of form I, cyan C atoms indicate subunit *B* of form I and magenta C atoms indicate form II. The catalytic Zn^2+^ is shown as a grey sphere and the catalytic water is shown as a red sphere. Dashed lines represent potential hydrogen-bonding interactions and the metal-ion coordination is depicted in continuous grey lines.

The deaminase domain of RibD has significantly more conserved residues than the reductase domain (Fig. 3 ▶). For example, the deaminase domains of *Ab*RibD and *Ec*RibD share 58% sequence identity, compared with 35% for the reductase domain and 46% overall. The Zn^2+^-binding site of the deaminase domain is located at the base of an opening in the structure lined with conserved residues (Fig. 4 ▶). Although no extra Zn^2+^ was present in the bacterial culture or during purification of the protein, well defined electron density with an OMIT difference density peak height of over 30σ compared with 7σ for a well defined carbonyl O atom was observed in the active site. This is consistent with the presence of Zn^2+^. The ion displays tetrahedral coordination by a conserved zinc-binding motif comprising His54, Cys79 and Cys88 and a water molecule in form I. An additional strong electron-density feature was observed coordinated to the Zn^2+^ in crystal form II. Examination of difference anomalous Fourier maps in similar fashion to nicotinamidase (data not shown; Fyfe *et al.*, 2009 ▶) suggested that this is cacodylate, which has replaced the water molecule. The peak height in an anomalous difference Fourier map was significantly higher than that of the cysteine S atoms, with a peak height of 8.5σ at the arsenic position compared with 4.5–5.6σ for sulfur. The *f*′′ signal is estimated to be 0.5 and 1.0 electrons for S and As^3+^, respectively, at a wavelength of 1.5418 Å.

Electron density consistent with the presence of an oxyanion binding to His54 was noted in both crystal forms. In form I this was assigned as phosphate since 0.9 *M* ammonium phosphate was used in crystallization and in form II as sulfate since 0.2 *M* ammonium sulfate was used in this case. The oxyanions, which represents the first ligands observed in the deaminase domain of RibD, interact with conserved residues and may represent the phosphate-binding site of the substrate.

The interactions of the phosphate group differ in subunits *A* and *B* (Fig. 4 ▶). In subunit *A*, the phosphate accepts hydrogen bonds from the side chains of Asn28, His54, His81, Thr85 and the main-chain amide of Arg84. In subunit *B*, the side chain of His81 points away from the active site, while the side chain of Arg84 is now within interaction distance of the phosphate. In form II, the side chain of Arg84 is oriented away from the active site, while His81 adopts a conformation intermediate between the two observed in form I. All residues interacting with the oxyanions are strictly conserved in RibD sequences, with the exception of Arg84, which is sometimes replaced by lysine (Fig. 3 ▶). Indeed, mutation of Lys79 in *Bs*RibG, which corresponds to Arg84 in *Ab*RibD, to alanine abolished deaminase activity (Chen *et al.*, 2009 ▶).

His81, Arg84 and Thr85 form part of the canonical RibD Zn^2+^-binding sequence PC*X*H*X*G(R/K)TPPC. The Zn^2+^ coordination sphere involves His54, Cys79, Cys88 and a water molecule in the apo form. Asn28 forms a hydrogen bond to the highly conserved His47 to form part of the substrate-binding site. A twofold to threefold reduction in activity was observed in *Bs*RibG when the corresponding residues were individually mutated to alanine (Chen *et al.*, 2006 ▶). These residues have been proven to be essential for binding substrate and may interact with the 5-amino and ribose groups, respectively (Magalhães *et al.*, 2008 ▶). The substrate pyrimidine is likely to bind sandwiched between His54 on one side and Pro29 and Leu76 on the other, with the side chains of Asn28 and His47 providing hydrogen-bonding capacity to assist this placement. Positioned at the other side of the deaminase active site are the catalytic Zn^2+^ and Glu56. These may act in concert to activate a water to generate a nucleophilic hydroxide that is then able to attack the substrate in a similar fashion as proposed for cytidine deaminase (see, for example, Ko *et al.*, 2003 ▶).

The loop between β4 and α4 differs in position in subunits *A* and *B* (crystal form I). This is a well conserved sequence that includes Asp106 (present as glutamate in a few sequences). This side chain may assist catalysis by interacting with the ‘leaving’ amine, so the different positions of this loop may possibly represent ‘open’ and ‘closed’ conformations of the active site. They certainly indicate that there is conformational freedom in this part of the structure.

Closely related structures were identified using the *Secondary Structure Matching* (*SSM*) server (Krissinel & Henrick, 2004 ▶). A search using the N-terminal domain alone matched members of the cytidine deaminase superfamily, with r.m.s.d.s in the range 1.3–1.8 Å for approximately 110 aligned C^α^ atoms. This structural similarity gives some insight into the mechanism of this domain, as other members of this superfamily have been extensively studied. The main-chain carbonyl of Thr127 in *E. coli* cytidine deaminase (PDB entry 1af2) is thought to form a leaving-group pocket together with Pro128 (Xiang *et al.*, 1997 ▶). The proline is conserved in RibD (Pro78); it is in fact part of the canonical motif described above. The threonine is replaced by glutamic acid (residue 77 in *Ab*RibD, strictly conserved in RibD sequences), with the side chain locked into position by interactions with the main-chain atoms of residues in α4. Given the structural similarities of the enzymes, it may be instructive to test whether cytidine deaminase inhibitors (see, for example, Ludek *et al.*, 2009 ▶) could provide templates for the development of RibD inhibitors.

### 3.4. The reductase domain

The active site in the reductase domain is located approximately 30 Å from the Zn^2+^-binding site on the opposite side of the subunit from the deaminase domain (Fig. 2 ▶ *b*). Our attempts at cocrystallization and soaking crystals with ligands targeted to bind in the reductase active site, for example NADPH, were unsuccessful (data not shown). Complex structures obtained by cocrystallization and soaking experiments in other laboratories showed that the cofactor NADPH binds in a long surface-exposed groove between α8 and α11 (see, for example, Stenmark *et al.*, 2007 ▶). There is significant variation in the orientation of the adenine with respect to the rest of the ligand, possibly reflecting the limitations of soaking experiments and the comparatively low resolution of the analyses. In both cases the resolution was 3.0 Å. The NADPH–*Bs*RibG structure (PDB entry 2d5n; S.-J. Chen, Y.-C. Chang & S.-H. Liaw, unpublished work) displayed NADPH binding in only one of the four molecules in the asymmetric unit, while in NADPH–*Ec*RibD (PDB entry 2o7p; Structural Proteomics in Europe, unpublished work) part of the nicotinamide-binding region was disordered in one subunit and the orientation of the nicotinamide group varied significantly between the subunits. This observation may be explained by difficulties in modelling the electron density of the nicotinamide ring close to the side chain of a tryptophan (Trp170*B*) at 3 Å resolution.

The substrate-binding site is between two loops. The first joins β6 to α7, which caps one end of the active site (Fig. 2 ▶ *a*). The reductase domain has previously been identified as being structurally similar to dihydrofolate reductase (DHFR; Chen *et al.*, 2009 ▶) and the β6–α7 loop overlays with the Met20 loop in *E. coli* DHFR, which undergoes large conformational changes throughout the catalytic cycle (Sawaya & Kraut, 1997 ▶). The second, very long, loop joins α8 to β8 (*via* a 3_10_-­helical section).

**Figure 5:**
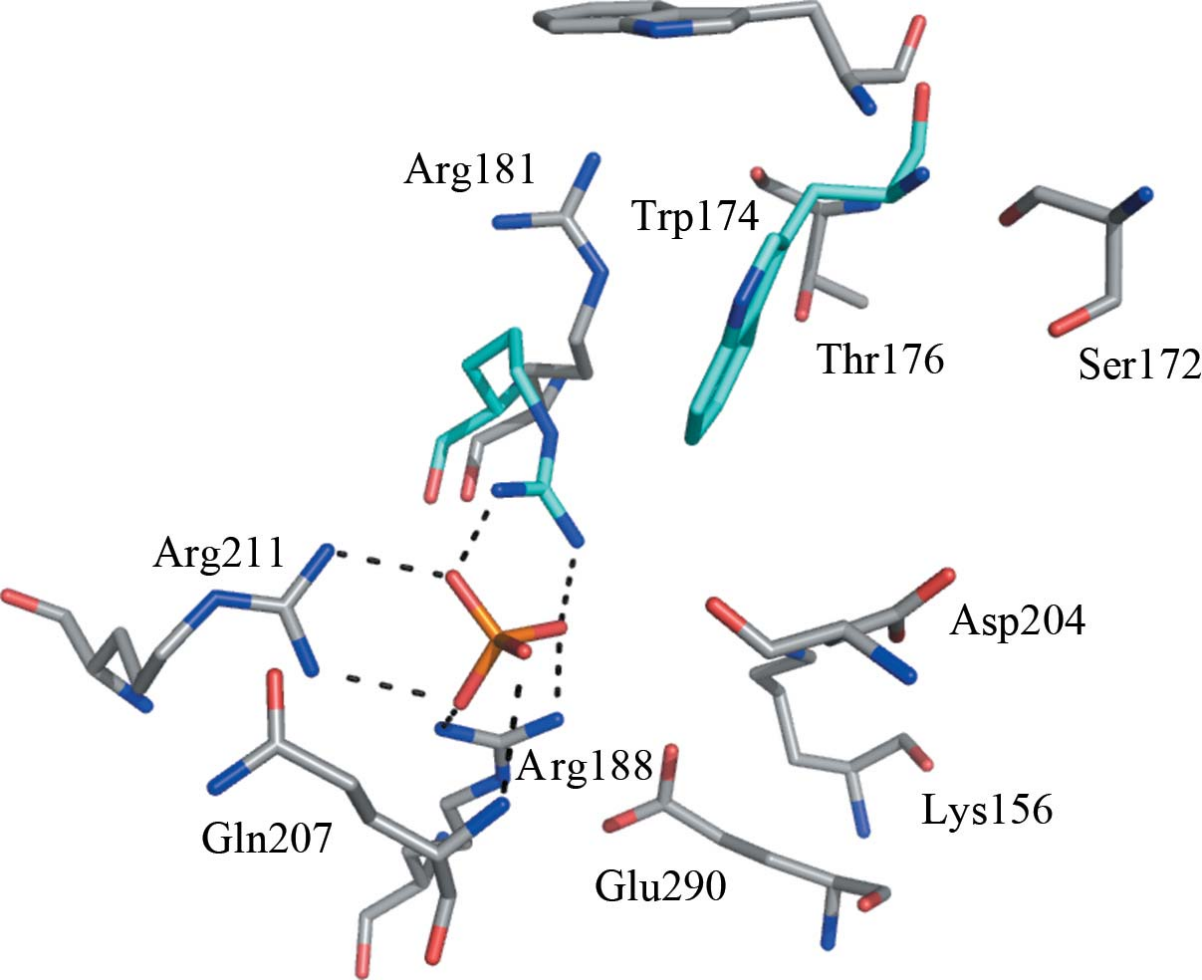
The reductase active site. The same colour scheme is used as in Fig. 4 ▶.

In similar fashion to what was observed in the deaminase active site, the oxyanions phosphate and sulfate were identified in the reductase active sites in forms I and II, respectively. These anions occupy the same position as the phosphate groups of the soaked ligands reported in the *Ec*RibD and *Bs*RibD structures. The phosphate interacts with the side chains of Arg188 and Arg211 and the main chain of Gln207. In subunit *B*, an additional interaction is formed with Arg181 (Fig. 5 ▶). A difference in the position of Arg181 in subunit *B* compared with subunit *A* is accompanied by movement of Trp174 into the active site, and a comparison with the substrate analogue binding position in *Bs*RibG (PDB entry 3ex8; Chen *et al.*, 2009 ▶) indicates this tryptophan is important in defining the active site. The pyrimidine moiety could fit well sandwiched between the NADPH nicotinamide and Trp174. This arginine–tryptophan pair is strongly conserved in RibD sequences (Fig. 3 ▶). In crystal form II Trp174 is not visible in the electron density, suggesting a degree of flexibility in this part of the active site consistent with the altered positions noted in form I.

Other conserved residues in this active site include Lys156, Ser172, Thr176, Asp204 and Glu290 (Figs. 2 ▶ and 5 ▶). Thr176 is well placed to interact with an amino group on the pyrimidine ring of the substrate. Based on the structure of *Bs*RibG with product, Chen *et al.* (2009 ▶) suggested that Glu290 (also Glu290 in *Ab*RibD) initiates the reaction, while Stenmark *et al.* (2007 ▶) identified the corresponding residue as Asp204 in *Ec*RibD. Further work would be required to address this point.

**Figure 6:**
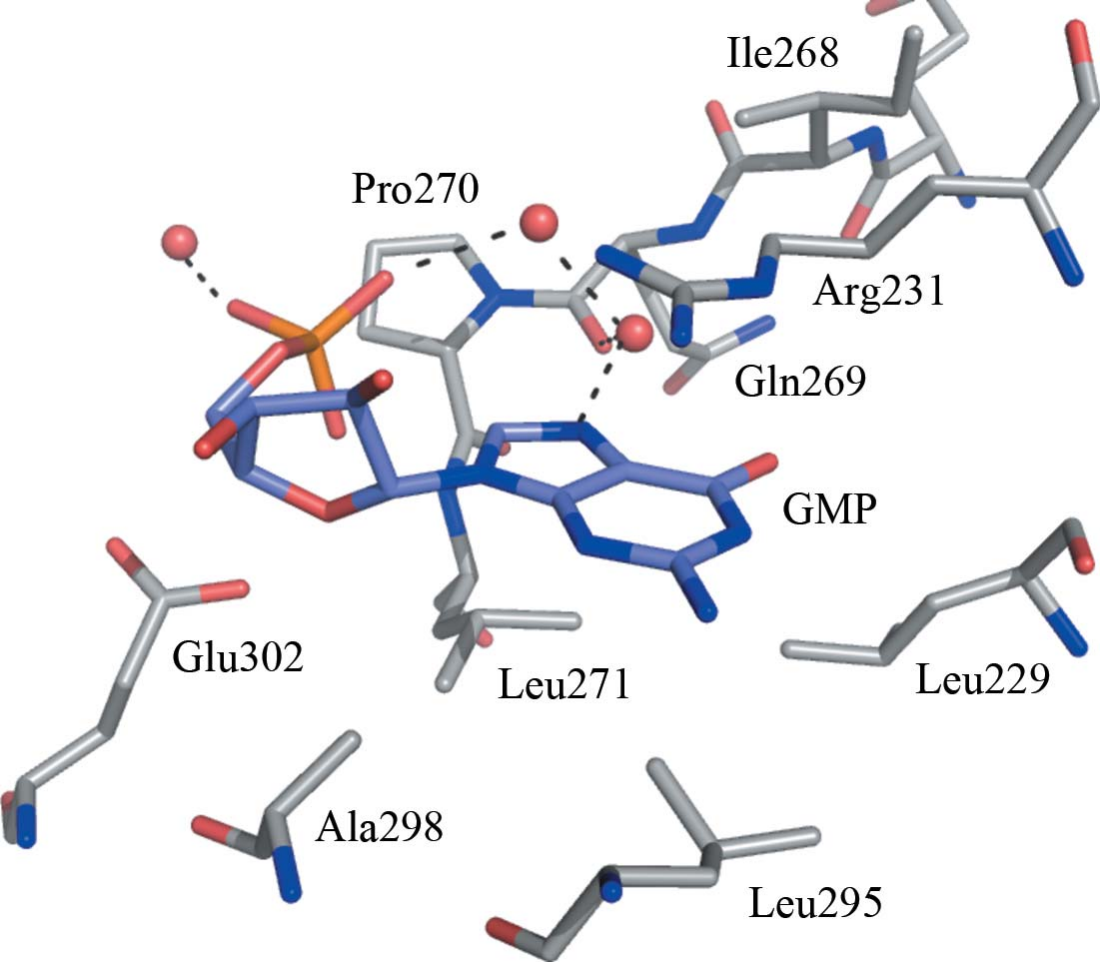
GMP binding in the adenosine-binding pocket. The same colour scheme is used as in Fig. 4 ▶ and in addition the C atoms of GMP are coloured slate blue.

Crystal form II was obtained in the presence of 5 m*M* GMP, which had been added as a potential substrate analogue for the deaminase reaction. Unexpectedly, a GMP molecule was observed in the NADPH-binding site of the reductase domain, occupying the adenine-binding pocket (Fig. 6 ▶). There are no direct hydrogen bonds involving the purine moiety and the enzyme; rather, it is positioned by van der Waals interactions with Leu271 and Leu295 on one side and the side chain of Arg231 on the other. Other residues that help to construct this cavity include Ala298, Glu302, Leu229, Gln269, Ile268 and Pro270.

A second GMP, refined with an occupancy of 0.5, was observed positioned in a depression on the surface of the protein formed between α7 and β14 (data not shown) and distant from either active site. Binding interactions appear to be dominated by van der Waals interactions between the purine and His186 and Trp187. We do not think that the GMP binding we observe is of biological relevance; rather, it is a consequence of the crystallization conditions with the high level of GMP present.

SSM analysis using the reductase domain identified the closest match as the equivalent monofunctional protein from *M. jannaschii* (PDB entry 2azn; Chatwell *et al.*, 2006 ▶) with an r.m.s.d. of 1.5 Å over 194 C^α^ atoms. The next closest are various type I DHFR proteins, with an r.m.s.d. range of 1.8–2.2 Å over approximately 150 C^α^ atoms. The closest matching DHFR is from *T. maritima* (PDB entry 1cz3; Dams *et al.*, 2000 ▶), with an r.m.s.d. of 1.7 Å over 152 C^α^ atoms, a *Z*-­score of 11.7 and a sequence identity of 22%; this DHFR does not have the flexible active-site loop seen in other DHFR structures.

## 4. Concluding remarks

An efficient recombinant expression system for the potential antibacterial drug target *Ab*RibD has been obtained and protocols have been established for purification and crystallization. Two ordered crystal forms have been obtained and the scene is set for a structure-based approach to ligand discovery. Given the structural and mechanistic similarities between RibD, cytidine deaminase and DHFR, it may be possible to exploit known inhibitors to provide starting points relevant to antibacterial drug discovery.

## Supplementary Material

PDB reference: RibD, 3zpc

PDB reference: 3zpg

## Figures and Tables

**Table 1:** Crystallographic statistics Values in parentheses are for the highest resolution shell.

	Form I	Form II
Resolution range (Å)	53.34–2.20	51.62–1.99
Unit-cell parameters (Å)	*a* = 148.99, *b* = 189.55, *c* = 76.41	*a* = *b* = 73.00, *c* = 201.46
Space group	*C*222_1_	*P*4_3_2_1_2
Measured reflections	159099 (22070)	504963 (63390)
Unique reflections	54441 (7963)	38040 (5291)
Multiplicity	2.9 (2.8)	13.3 (12.0)
Wilson *B* (Å^2^)	34.2	33.0
Completeness (%)	99.0 (99.7)	99.5 (96.1)
〈*I*/σ(*I*)〉	6.4 (1.9)	24.7 (4.1)
*R*_merge_ †	0.092 (0.439)	0.064 (0.622)
No. of protein residues (chain *A*/*B*)	357/356	354
No. of waters	176	127
No. of Zn^2+^ ions	2	1
No. of acetate ions	1	1
No. of phosphate ions	4	—
No. of sulfate ions	—	2
No. of oxalate ions	—	2
No. of cacodylate ions	—	1
No. of Cl^−^ ions	—	1
No. of GMP molecules	—	2
*R*_work_ ‡/*R*_free_ § (%)	21.28/25.04	21.40/23.67
DPI¶ (Å)	0.16	0.12
Average *B* factors (Å^2^)		
Overall (chain *A*/*B*)	37.2/46.2	44.78
Side chain (chain *A*/*B*)	39.2/49.3	45.73
Main chain (chain *A*/*B*)	37.2/47.7	43.86
Water	35.7	40.3
Zn^2+^	32.6	54.5
Acetate	30.8	34.0
Phosphate	39.4	—
Sulfate	—	65.5
Oxalate	—	53.4
Cacodylate	—	50.2
Cl^−^	—	33.82
GMP	—	59.48
R.m.s.d., bond lengths (Å)	0.011	0.010
R.m.s.d., bond angles (°)	1.30	1.29
Ramachandran plot analysis†† (%)		
Favourable	97.2	97.4
Outliers	0.0	0.3 [Gly170]

† *R*_merge_ = 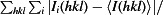 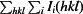, where *I* *_i_*(*hkl*) is the intensity of the *i*th measurement of reflection *hkl* and 〈*I*(*hkl*)〉 is the mean value of *I_i_*(*hkl*) for all *i* measurements.

‡ *R*_work_ = 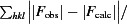 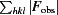, where *F*_obs_ is the observed structure-factor amplitude and the *F*_calc_ is the structure-factor amplitude calculated from the model.

§ *R*_free_ is the same as *R*_work_ except calculated with a subset (5%) of data that were excluded from refinement calculations.

¶ The diffraction-component precision index (DPI) as defined by Cruickshank (1999 ▶)

†† According to *MolProbity* (Chen *et al.*, 2010 ▶).
